# Comparing multiple statistical software for multiple-indicator, multiple-cause modeling: an application of gender disparity in adult cognitive functioning using MIDUS II dataset

**DOI:** 10.1186/s12874-020-01150-4

**Published:** 2020-11-12

**Authors:** Chi Chang, Joseph Gardiner, Richard Houang, Yan-Liang Yu

**Affiliations:** 1 Office of Medical Education Research and Development, College of Human Medicine, Michigan State University, 965 Wilson Rd., Room A214C, East Lansing, MI 48824 USA; 2 Department of Epidemiology and Biostatistics, College of Human Medicine, Michigan State University, East Lansing, MI 48824 USA; 3 Center for the Study of Curriculum Policy, College of Education, Michigan State University, East Lansing, MI 48824 USA; 4 Department of Sociology and Criminology, Howard University, Washington, DC 20059 USA

**Keywords:** MIMIC model, MIDUS II, Statistical software package comparison, Cognitive functioning performance, Latent variable framework, Structural equation model, R, M*plus*, SAS

## Abstract

**Background:**

The multiple-indicator, multiple-cause model (MIMIC) incorporates covariates of interest in the factor analysis. It is a special case of structural equation modeling (SEM), which is modeled under latent variable framework. The MIMIC model provides rigorous results and becomes broadly available in multiple statistical software. The current study introduces the MIMIC model and how it can be implemented using statistical software packages SAS CALIS procedure, R *lavaan* package, and M*plus* version 8.0.

**Methods:**

In this paper, we first discussed the formulation of the MIMIC model with regard to model specification and identification. We then demonstrated the empirical application of the MIMIC model with the Midlife in the United States II (MIDUS II) Study (*N* = 4109) using SAS CALIS procedure, R *lavaan* package and M*plus* version 8.0 to examine gender disparities in cognitive functioning. The input, output, and diagram syntaxes of the three statistical software packages were also presented.

**Results:**

In terms of data structure, all three statistical programs can be conducted using both raw data and empirical covariance matrix. SAS and R are comprehensive statistical analytic packages and encompass numerous data manipulation capacities. M*plus* is designed primarily for latent variable modeling and has far more modeling flexibility compared to SAS and R, but limited in data manipulation. Differences in model results from the three statistical programs are trivial. Overall, the results show that while men show better performance in executive function than women, women demonstrate better episodic memory than men.

**Conclusions:**

Our study demonstrates the utility of the MIMIC model in its empirical application, fitted with three popular statistical software packages. Results from our models align with empirical findings from previous research. We provide coding procedures and examples with detailed explanations in the hopes of providing a concise tutorial for researchers and methodologists interested in incorporating latent constructs with multiple indicators and multiple covariates in their research projects. Future researchers are encouraged to adopt this flexible and rigorous modeling approach.

## Background

This paper illustrates how to implement multiple-indicator, multiple-cause (MIMIC) modeling, a special case of structural equation model (SEM), under the latent variable modeling (LVM) framework using three statistical software packages: SAS CALIS procedure, M*plus*, and R *lavaan* package. SAS is widely used in health sciences and M*plus* is commonly used in social sciences for LVM. R has become popular in recent years because it is open source and free. In addition, all three packages generate path (or structural or causal) diagrams to help interpreting the output. This paper consists of two sections: 1) introduction of the MIMIC model, and 2) illustration of fitting the MIMIC model to cognitive function theory using the MIDUS II dataset [1].

Two types of variables are generally encountered in research: observed and unobserved. Observed variables are also referred to as manifest variables. For example, gender, age, responses to questions in surveys, and ranked observations by raters are examples of manifest variables. Unobserved variables can be regarded as latent constructs; examples include anxiety, quality of life, or sickness. To understand latent constructs, researchers rely on observed or measured variables. Therefore, the measured variables are also called measured indicators. For example, because of its multifaceted domains, the quality of life of an individual cannot be directly observed. Several indicators could describe quality of life. As a latent construct, it can be measured in different domains and by researchers’ values and perspectives on this latent concept. Measured aspects can include, but are not limited to, “the number of days in a week that one feels stressed”, “the number of days in a month one needs to worry about money”, or “the number of weeks this year one has to take care of parents.” Therefore, when it comes to deciding on indicators for measuring a latent construct, it is important that researchers have theoretical background knowledge to narrow down the range of perspectives and to focus on the definition of the construct and its use in a to-be-tested model. Researchers’ content knowledge is also essential in the model modification step, which will be discussed below.

SEM combines both measurement and structural considerations. It integrates psychometric concepts (i.e., measurement approaches) and the econometric ideas (structure approaches). The aforementioned examples in which the latent construct (i.e., health, sickness, quality of life, anxiety) is measured by indicators is regarded as the measurement approach to SEM. Measurement errors of indicators are taken into consideration. As for the structure approaches in SEM, path analysis is applied to estimate the relationships among latent constructs. The ability to combine these two analyses is one of the advantages of SEM. By specifying and describing the plausible relationships between latent concepts and manifest variables, associated measurement errors and proposed structural relationships among latent structures in SEM can effectively estimate parameters simultaneously, which mirror the fact that the variables coexist in reality [2].

Another advantage of SEM is that the measurement model in the latent variable approach takes into account potential measurement errors of the indicators. Traditional multiple regression analysis assumes that the independent variables included in the model are error-free. However, if this assumption is not tenable, it will result in biased estimates of the regression coefficient and incorrect conclusions. SEM incorporates measurement errors during construction of the latent variables while simultaneously estimating the relationships among those latent variables, making this approach powerful and flexible.

Due to its broad capabilities and application in a diversity of fields, the emerging popularity of SEM has led to the development of statistical software packages for analysis based on SEM. Currently, available software packages include M*plus*, STATA, LISREL, EQS, AMOS, *lavaan* package in R, and SAS CALIS procedure. Among these software, SAS is commonly used in biostatistics area and pharmaceutical companies; R is a free statistical computing language known for its rich packages; M*plus* is especially designed for running latent variable models and is commonly used in social science field. Therefore, this paper focuses on using SAS CALIS procedure, R *lavaan* package, and M*plus*. We begin with a brief introduction to the fundamental concepts of SEM, followed by a special case of SEM, the MIMIC model. The MIMIC model is popular in epidemiology on how to contextualize latent variables of interest. In this paper, we use a dataset of 4109 participants in MIDUS II to demonstrate how the MIMIC model can be used to examine gender disparity in cognitive functions via the three statistical software programs. Their input codes for generating the path diagram are also provided in this paper.

Since the purpose of this paper is to provide a tutorial of modeling procedure in fitting the MIMIC model with different software, answering the empirical questions in the development of the cognitive theory and gender disparity in cognitive function are not the focus of this paper. The measurement part of the MIMIC model in our empirical example is based on the factor analysis in Lachman, Agrigoroaei, Tun, & Weaver [3]. More detailed discussions on cognitive functioning and the psychometric properties of the instruments used in MIDUS II can be found in Lachman et al’s study [3]. Details of the technique of SEM (e.g., model estimation and model evaluation) in general, and MIMIC in particular, can be found in Bollen & Long [4]; Jöreskog & Goldberger [5]; O’Rourke & Hatcher [6]; and Wang & Wang [7].

## Methods

In this section, we discussed the formulation of the MIMIC model with regard to model specification, model identification, and model fit. We started from introducing the components in one-factor MIMIC model and extended it to multiple-factor MIMIC model. Detailed assumptions of the MIMIC model and how to calculate the number of free parameters to construct a just-identified or over-identified model were elaborated. In the results section, we demonstrated the application of the two-factor MIMIC model with a real dataset from the Midlife in the United States II (MIDUS II) Study (*N* = 4109) using SAS CALIS procedure, R *lavaan* package and M*plus* version 8.0 to examine gender disparities in cognitive functioning. The input, output, and diagram syntaxes of the three statistical software programs were also presented respectively. The parameter estimates results were compared, the interpretation, and the detailed reference of each software package were provided for practitioners.

### MIMIC model

#### Model specification

The Multiple Indicators, Multiple Causes Model (MIMIC) is an extension of confirmatory factor analysis (CFA) with covariates. This model is commonly used to contextualize the latent variables of interest (e.g., quality of life, motor ability) using the demographic variables (e.g., age, gender), and it is assumed that those demographic variables are measured without error. By incorporating these covariates in CFA under the latent variable framework, the relationships between the demographic variables and the latent variables of interest are simultaneously estimated with the factor loadings in the measurement model. It is known for its advantage in simultaneous estimation of parameters. In addition, including direct paths from the demographic variables to the indicators of the latent variable would allow for examining differential item functioning effects for each indicator [8]. The modeling strategy provides an alternative way in the validation process of the examination when psychometric properties of the instrument/scale are the research interest, in which items with gender disparities or racial disparities can be identified [9–11]. The demographic variables are the ‘cause’ variables in the model. However, since there is no implication of causal effect in the model, it is also called exogenous manifest variables (in the field of econometrics), or predictors (in the field of psychology), to avoid confusion and to differentiate it from the indicators in the measurement model. Note that any type of covariates (i.e., continuous or categorical) can be included as the exogenous manifest variables in a MIMIC model.

Path diagrams are commonly used in LVM to depict the measurement and structural equations with a pictorial representation. In the diagram, we use squares to represent observed variables and circles to represent latent or unobserved variables. Arrows are used to indicate directed relationships. An arrow from X to Y represents a linear relationship in which Y is the dependent variable and X is the independent variable. The diagrams map out the relationships among constructs and covariates, facilitating the discussion and therefore, is one of the key features of the latent variable modeling.

A general linear structural relation (LISREL) model [12] consists of a set of linear structural equations with two parts: the measurement model and the structural model. The measurement model specifies how the unobserved latent constructs were measured by the indicators, and the structural model specifies the relationships between the latent variables. Since it allows for estimating measurement error variances for the measurement model and the disturbance variance-covariance matrix for the structural part, as well as the unknown coefficient among the structural relations [12], LISREL models gains great interest and attention from interdisciplinary fields. Latent variable modeling was later used interchangeably with LISREL to emphasize that the general LISREL model can also be used in modeling nonlinear relationships between unobserved and observed variables.

**Fig. 1:**
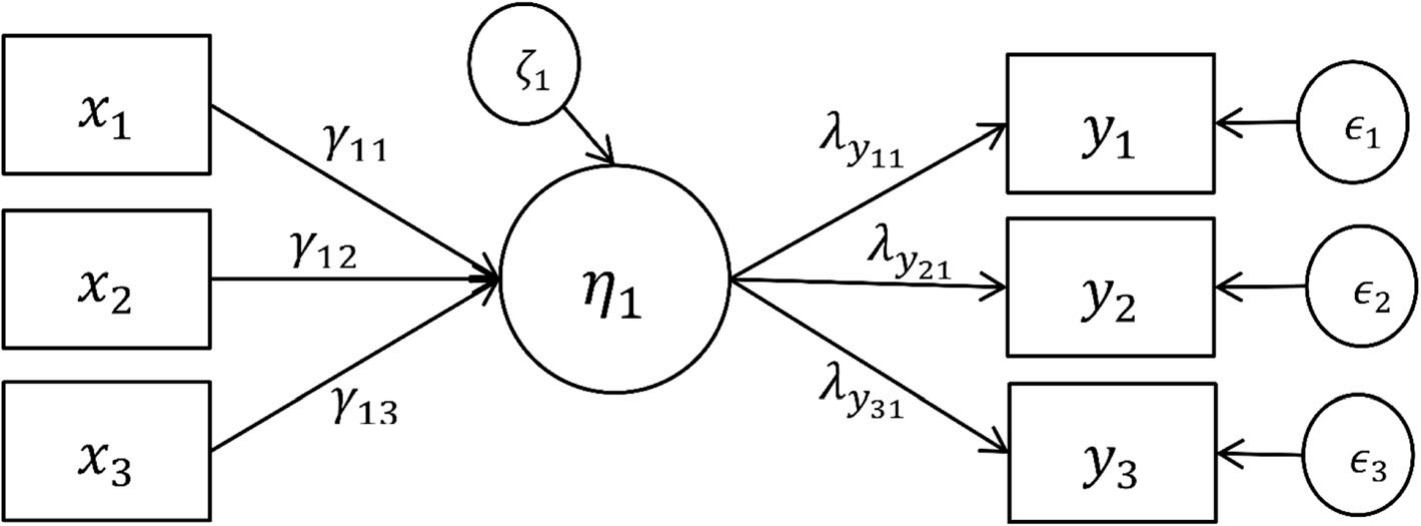
A one-factor MIMIC model

MIMIC model is a special case of SEM. For the sake of simplicity in this study, the model is first illustrated by a one-factor linear MIMIC model scenario (See Fig. 1). The measurement part is a general LISREL model for a *p* × 1 vector **Y** of endogenous variables followed by the structural part that incorporates the influence of exogenous variables, denoted by the *q* × 1 vector **X**, where *p* indicates the number of measured indicators, and *q* indicates the number of predictors. The single latent factor ties the two parts,

$$ \boldsymbol{Y}=\boldsymbol{\Lambda} \eta +\epsilon $$

$$ \eta ={\boldsymbol{\Gamma}}^{\prime}\boldsymbol{X}+\zeta $$

**Y** is also called measured indicators, and **X** referred to as predictors (i.e., exogenous manifest variables or cause variables). The *p* × 1 vector **Λ** and the *q* × 1 vector **Γ** are regression coefficients, or path coefficients. *ϵ* is the residual or measurement errors, and *ζ* is the disturbances. In other words, *ζ* is the error term for the regression of *η* on the covariates (***X***). The reduced form of (1) is expressed as

$$ \boldsymbol{Y}=\boldsymbol{\Lambda} \left({\boldsymbol{\Gamma}}^{\prime}\boldsymbol{X}+\zeta \right)+\epsilon $$

$$ =\boldsymbol{\Lambda} {\boldsymbol{\Gamma}}^{\prime}\boldsymbol{X}+\left(\boldsymbol{\Lambda} \zeta +\epsilon \right) $$

$$ =\Pi \boldsymbol{X}+\omega $$

where Π = ΛΓ′ and *ω* = **Λ***ζ* + *ϵ* is the composite error.

The standard assumptions of the MIMIC model are: (1) *ϵ* is uncorrelated with, and (2) *ϵ*, *ζ* are uncorrelated with **X**. These assumptions are sufficient to derive the covariance matrix of **Y**. From (2), $\mathit{\operatorname{cov}}\left(\boldsymbol{Y}\right)=\mathbf{\prod}{\boldsymbol{\Theta}}_{\boldsymbol{X}}{\mathbf{\prod}}^{\prime }+\lambda {\lambda}^{\prime }{\sigma}_{\zeta}^2+{\boldsymbol{\Theta}}_{\boldsymbol{\epsilon}}$, where **Θ**_**X**_ ***=*** *Cov*(***X***), **Θ**_***ϵ***_ = *Cov*(*ϵ*), ${\sigma}_{\zeta}^2= Var\left(\zeta \right)$. For model identification, **Θ**_***ϵ***_ is diagonal. The one-factor model has 2*p* + *q* + 1 parameters. Note that model specification does not include a mean structure.

A multiple-factor MIMIC model has *m* latent variables. Replace (1) by.

1 $$ \boldsymbol{Y}=\boldsymbol{\Lambda} \boldsymbol{\eta} +\boldsymbol{\epsilon} $$

2 $$ \boldsymbol{\eta} =\boldsymbol{B}{\boldsymbol{\eta}}^{\prime }+\boldsymbol{\Gamma} \boldsymbol{X}+\zeta $$

Let *m* be the number of latent variables, **Λ** is a *p* × *m* matrix of regression coefficients of **Y** on ***η***, **Γ** is a *m* × *q* matrix of regression coefficients of ***η*** on **X**. The *m* × *m* matrix ***B*** are the regression coefficients of variables in ***η*** on other variables in ***η***. The diagonal elements of ***B*** are zero, but (***I − B***) is assumed invertible.

The reduced form of (3) is **Y** = **Λ*η*** + ***ϵ*** = **Λ**[***I − B***]^***−*1**^**Γ*X +*** **Λ**[***I − B***]^***−*1**^***ζ + ϵ*** from which we get an expression for the covariance of **Y**, and the covariance between **Y**, **X** under the assumptions: (1) ***ϵ*** is uncorrelated with ***ζ*** and (2) ***ϵ***, ***ζ*** are uncorrelated with **X**.

$$ Cov\left(\boldsymbol{Y}\right)=\boldsymbol{\Lambda} {\left[\boldsymbol{I}-\boldsymbol{B}\right]}^{-\mathbf{1}}\left(\boldsymbol{\Gamma} {\boldsymbol{\Theta}}_{\boldsymbol{X}}{\boldsymbol{\Gamma}}^{\prime }+\boldsymbol{\Psi} \right){\left[\boldsymbol{I}-\boldsymbol{B}\right]}^{-\mathbf{1}}{\boldsymbol{\Lambda}}^{\prime }+{\boldsymbol{\Theta}}_{\boldsymbol{\epsilon}} $$

$$ Cov\left(\boldsymbol{Y},\boldsymbol{X}\right)=\boldsymbol{\Lambda} {\left[\boldsymbol{I}-\boldsymbol{B}\right]}^{-\mathbf{1}}\boldsymbol{\Gamma} {\boldsymbol{\Theta}}_{\boldsymbol{X}} $$

where **Ψ** ***=*** *Cov*(**ζ**). The input data set for estimation of parameters in (**Λ,Γ,B**) and in (**Ψ**, **Θ**_***ϵ***_) is an empirical correlation or covariance matrix ***S*** for the manifest variables **Y**, **X**. A standard estimation method is maximum likelihood, which optimizes the distance between ***S*** and the model-induced variance-covariance matrix: **Σ**. There are $\frac{1}{2}\left(p+q\right)\left(p+q+1\right)$ data elements in **Σ**. The model has variance-covariance parameters in **Σ**, while have *q* × *m* regression coefficients. An example of a two-factor model is illustrated in the next section.

#### Model identification

To solve the scale indeterminacy issue in SEM, researchers can either set ${\sigma}_{\zeta}^2=1$ or one of the λ's equal to a certain value. Here MIMIC model is applied as an example.

Suppose the disturbance variable (*ζ*) is standardized. Distinct elements of parameters in the variance-covariance matrix of *q* exogenous variables (X) and *p* measured indicators (Y): $\frac{\left(p+q\right)\times \left(p+q+1\right)}{2}$. In MIMIC model, the to-be-estimated parameters include: 1) *p* factor loadings (*λ*_1_…*λ*_*p*_)), 2) *p* nonzero residual variances ${\sigma}_{\epsilon_1}^2$, …, ${\sigma}_{\epsilon_p}^2$, 3) *q* × *m* regression coefficients (*γ*), where *m* is the number of latent constructs, 4) *m* latent construct residual variances (or the disturbance variable, *ζ*), which equals zero to solve for scale indeterminacy, 5) $\frac{m\left(m-1\right)}{2}$ covariance (**Ψ**) between residual variances (*ζ*) of the latent constructs, and 6) $\frac{q\left(q+1\right)}{2}$ variances and covariances among exogenous variables (or predictors). Based on the counting rule, if the number of to-be estimated parameters (i.e., the number of free parameters) is equal to or less than the number of nonredundant elements in population covariance matrix **Σ**, then the model is called just identified or overidentified, respectively. Otherwise, the model can be not identified [13]. Based on the notations above, the one factor model can be extended to a multiple-factor model. In the following section, an example of a two-factor MIMIC model is illustrated.

#### Model fit

Model fit indicates the degree to which a model can reproduce the data. Though a good-fitting model is what a researcher pursues for, it is worth noting that a good-fitting model does not guarantee sensible and reasonable parameter estimates or a correctly specified model [14]. In other words, nonsensical results or poor validity evidence can be found from a good-fitting model. A reasonable model should consist of reasonable parameter estimates and good-fitting model fit. Even for a good-fitting model, model modification can still improve the model [14].

Latent variable modeling researchers originally used *χ*^2^ test statistic to measure/quantify model fit; however, it is sensitive to large sample size. Methodologists developed numerous fit indices to adjust the *χ*^2^ test statistics with the information in the model, such as degrees of freedom, sample size, and/or the number of variables. Depending on the elements in the formula, fit indices in latent variable models can be categorized into three types [15]: 1) relative fit indices (also called incremental fit index): Bentler-Bonett Index (NFI) [16], Tucker Lewis Index (TLI), and Comparative Fit Index (also called Bentler Comparative Fit Index, CFI) [17]. 2) absolute fit indices: Root Mean Square Error of Approximation (RMSEA), Standardized Root Mean Square Residual (SRMR), and 3) parsimony fit indices: Parsimony Goodness-of-Fit Index (PGFI) and Akaike information Criterion (AIC).

Although each index was developed to rectify the problems of other indices, considerable controversy about which fit indices and what cut-off criteria to use is substantial. For reporting purposes, the common practice is that unless the proposed model is close to saturated model or the purpose is to compare models, one should report *χ*^2^ test statistics and choose one index from each type: relative fit and absolute fit indices, to measure the model fit.

### Real data set

The theoretical framework of cognitive function theory and its psychometric features in this paper are adapted from Lachman et al. [3] We use a dataset of 4109 subjects from the MIDUS (Midlife in the United States) study wave II collected between 2004 and 2006 [1]. The data that support the findings of this study are available in the Inter-university Consortium for Political and Social Research at 10.3886/ICPSR25281.v6, reference number 25281. These data were derived from the following resources available in the public domain: http://midus.wisc.edu/index.php. We applied the same cognitive function framework for a measurement model, but adding a structural model with exogenous subject variables, age and gender to influence two latent constructs, Executive Function and Episodic Memory. The MIDUS (BTACT) Battery consists of seven tasks. The first latent construct, Executive Function is measured by five tasks: 1) Stop and Go Switch Task (SGST), 2) 30 Seconds and Counting Task (NmCorr), 3) Number Series (NmSr), 4) Category Verbal Fluency (UniItemF), and 5) Backward Digit Span (DgtSpan). The second latent construct, Episodic Memory is measured by two tasks: 1) Delayed Word List Recall (UniItemD), and 2) Immediate Word List Recall (UniItemI). These seven manifest variables Y are the indicators in the measurement model. The descriptive statistics of variables in the dataset is in Table 1.

**Table 1:** Descriptive Statistics of Indicators and Covariates in the Data Set

Variable	Male (*n* = 1870)		Female (*n* = 2239)		min	max
	Mean	SD	Mean	SD		
Age	55.69	12.14	55.4	12.25	28	84
UniItemI	6.24	2.1	7.22	2.27	0	15
NmRepI	0.23	0.6	0.3	0.81	0	14
NmIntI	0.48	0.8	0.47	0.82	0	7
UniItemD	3.79	2.39	4.91	2.68	0	14
NmRepD	0.09	0.4	0.11	0.56	0	16
NmIntD	0.91	1.48	0.88	1.49	0	26
DgtSpan	4.96	1.5	5.03	1.51	0	8
UniItemF	19.24	6.15	18.46	6.03	0	42
NmRepF	0.31	0.7	0.32	0.68	0	8
NmIntF	0.05	0.56	0.04	0.48	0	16
NmSr	2.42	1.55	2.07	1.49	0	5
lstNm	60.25	11.7	63.57	10.54	1	99
NmErr	0.86	2.64	0.86	1.79	0	90
NmCorr	38.89	11.81	35.57	10.92	−13	90
SGST	−1.07	0.24	−1.12	0.31	−7	−0.2

**Fig. 2:**
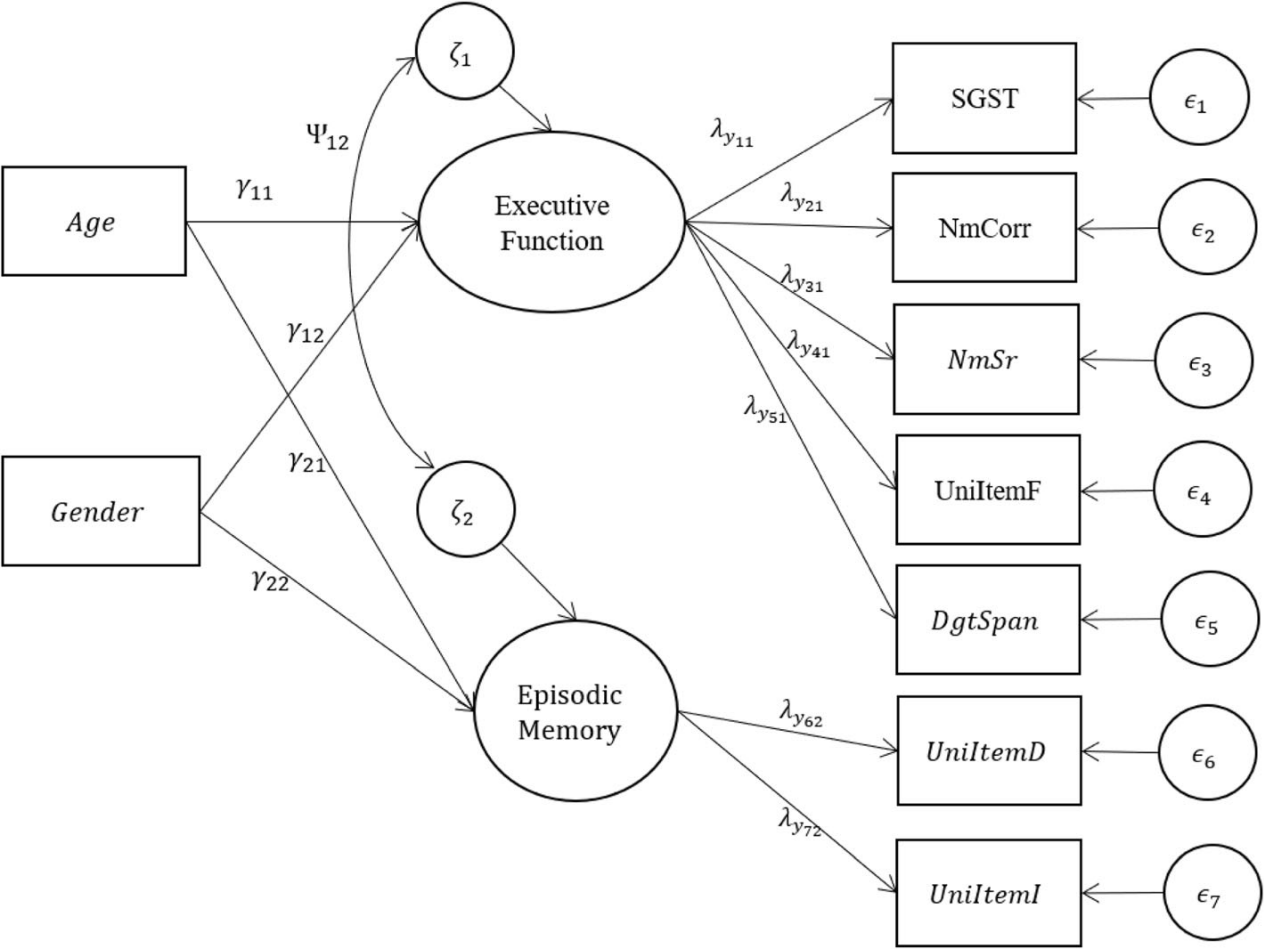
The path diagram of the MIMIC model - Initial Specification

*Note:* Paths indicated by single-headed arrows, alongside path coefficients. Errors (*ϵ*_1_, *ϵ*_2_, *ϵ*_3_, *ϵ*_4_, *ϵ*_5_, *ϵ*_6_, *ϵ*_7_) associated with manifest endogenous variables, and disturbances (*ζ*_1_, *ζ*_2_) associated with endogenous latent variables.

For the structural model for influences, two manifest variables (X) age and gender (=0 for men, =1 for women) are included as the predictors (i.e., the causes). Relationships between X and the two latent constructs, and their indicators Y in the MIMIC model are presented in Fig. 2.

## Results

### Empirical example

#### Model construction

The measurement model, ***Y =*** **Λ*η + ϵ***, are the following seven equations (see equation (1)):

$$ SGST={\lambda}_{11}{F}_1+{\epsilon}_1 $$

$$ mCorr={\lambda}_{21}{F}_1+{\epsilon}_2 $$

$$ mSr={\lambda}_{31}{F}_1+{\epsilon}_3 $$

$$ UnitItemF={\lambda}_{41}{F}_1+{\epsilon}_4 $$

$$ gtSpan={\lambda}_{51}{F}_1+{\epsilon}_5 $$

$$ UniItemD={\lambda}_{62}{F}_2+{\epsilon}_6 $$

$$ UniItemI={\lambda}_{72}{F}_2+{\epsilon}_7 $$

where ***η =*** (*F*_1_, *F*_2_)^′^ = (*Executive Function*, *Episodic Memory*)′. The matrix **Λ** of path coefficients has seven parameters. The seven parameters in the variance matrix, assumed diagonal, of the error *ϵ*. The structural model, ***η = Bη +*** **Γ*X + ζ*** is explicitly

$$ {F}_1={\gamma}_{11} Age+{\gamma}_{12} Gender+{\zeta}_1 $$

$$ {F}_2={\gamma}_{21} Age+{\gamma}_{22} Gender+{\zeta}_2 $$

where ***B*** indicated the variance covariance matrix among two latent constructs. In this model, there are predictors involved and the two latent constructs are the outcomes in this model. Therefore, ***B*** = 0, paths are not allowed between the two latent constructs. There are four path coefficients in **Γ**, and the covariance matrix of the disturbance *ζ* has a one covariance parameter **Ψ**_**12**_. Because the disturbance variance terms *ζ* are given a fixed value of 1 for identification, **Ψ**_**12**_ is also the correlation between two latent constructs. The simplified specification of equation (4) is *Cov*(***Y***) **= Λ**(**ΓΘ**_***X***_**Γ**^′^ ***+*** **Ψ**)**Λ**^′^ ***+*** **Θ**_***ϵ***_, *Cov*(***Y***, ***X***) = **ΛΓΘ**_***X***_, *Cov*(***X***) ***=*** **Θ**_***X***_. A total of 22 parameters in the specification (4) that must be estimated from the empirical covariance matrix ***S*** of (**Y**, **X**) that has 45 terms. The 22 to-be-estimated parameters include seven factor loadings (*λ*), seven residual variances of the indicators (*ϵ*), four regression coefficients between two causes and two latent structures (*γ*), two latent construct residual variances (*ζ*), one covariance (**Ψ**) between residual variances (*ζ*_1_, *ζ*_2_) of the latent constructs, and one covariance between two predictors. Therefore, the degrees of freedom of the proposed model is 45–22 = 23.

For scale indeterminacy, either one of the factor loadings or the residual variance of the latent structures should be fixed at 1. The covariance **Ψ**_**12**_ between the two residuals *ζ*_1_ and *ζ*_2_ is used to demonstrate the relationship between two latent constructs. If one chooses to fix the residual variances, *ζ*_1_ and *ζ*_2_, to 1, **Ψ**_**12**_ would be the correlation between the two constructs, like the example we demonstrate here.

In the following sections, we fit the proposed MIMIC model (shown in Fig. 2) to the MIDUS II dataset, which includes seven manifest variables (i.e., SGST, NmCor, NmSr, UnitItemF, DgtSpan, UnitItemD, and UnitItemI) and two predictors (or causes, i.e., age and gender). Explanations of the results as well as the input, output and diagram codes of SAS, R, and M*plus* are provided. Applied researchers can choose the software packages they prefer and adapt the syntax to their own data set.

### Software package comparison

#### SAS

In SAS, a MIMIC model can be fitted with the raw data set or the empirical covariance matrix S containing *Cov*(*Y*, *X*) using procedure CALIS. If one only has access to the S matrix, then the number of observation (NOBS option) needs to be specified after the name of the dataset in the syntax. In our example, if the data set is a covariance matrix, we specify “data = <<COVARIANCE MATRIX>>(type = cov) nobs = 4109” after PROC CALIS and before the modification option. The means of the variables are not needed. Both types of data input can produce the same results. Here, the SAS Input shows the syntax when raw data set is available. 

##### SAS input (Fig. 3)

the statements PATH, PVAR and PCOV are included in the SAS syntax to specify the parameters to be estimated. If the parameters specified are not model parameters, SAS will estimate them, too. Factor loadings and regression coefficients are specified in the PATH statement with a one-direction arrow. For example, “F1 ---> SGST” indicates that F1 is measured by SGST. Since F1 is not in the variable list, SAS reads it as a latent construct, specified and named F1, and measured by SGST. This is also how the factor loading is specified in the PATH statement. “age ---> F1” means regressing age on F1, which is the regression coefficient of age on the first latent factor requested to be estimated. The terms after the equal signs are the user-specified names for the parameters, which are optional. The PVAR statement specifies the variance and residual variances terms to be estimated with supplied names or given their fixed value. An alternative way to request variance estimation is to specify double-headed paths, “<− −>” and the variable in the path statement, then the PVAR statement can be skipped. For example, the syntax “<--> F1 1.0” in the PATH statement functions the same as “F1 = 1.0” in the PVAR statement, used to fix the residual variance of the F1 latent structure at 1. The syntax “<−- > SGST” requests the residual variance estimate of SGST. The syntax “<−- > age” requests the variance of the age variable. In SAS, all other variances will be estimated by default-- in this case the exogenous manifest variables age and gender.

**Fig. 3:**
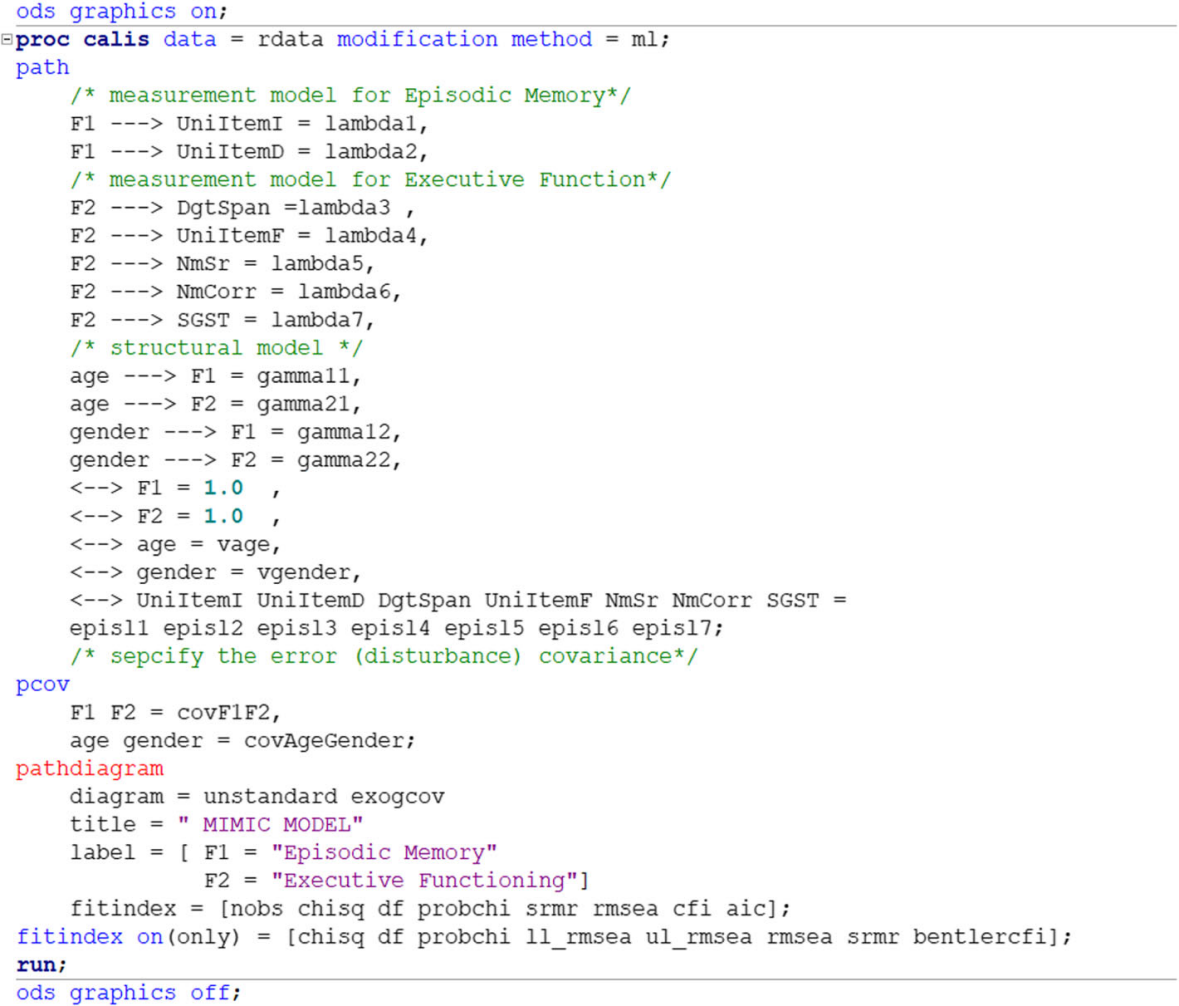
SAS syntax for the MIMIC model

The statement PCOV is used to specify the covariance terms and is the subsidiary model specification statement for the path model. For both PVAR and PCOV statements, the equal signs and the terms after the signs are the user-specified names for the parameters, which are optional. The PCOV statement, *F*1 *F*2 = *covF*1*F*2, names the covariance between F1 and F2 as “*covF1F2*”, and the parameter **Ψ**_**12**_ is to be estimated. Noted that the variance of age and gender requested in the PVAR statement and the covariance between age and gender requested in the PCOV statement are not model parameters. The three values in the output are directly from the covariance matrix S. Also noted, since two latent structures are used as the dependent variable in the structure model, when we specify the “F1 = 1.0” and “F2 = 1.0”, it is the residual variances of the latent constructs, ${\sigma}_{\zeta_1}^2$ and ${\sigma}_{\zeta_2}^2$ fixed at 1, rather than the variance of the latent constructs themselves. By default, PROC CALIS will estimate the following free parameters if not given a fixed value: error variances of all manifest or latent variables, and variances and covariance of all exogenous variables, manifest or latent. Note that although the variance and covariance of exogenous manifest variables are parameters to be estimated, they are not model parameters. The values showed in the result are directly from the sample variance-covariate matrix [13].

A PATHDIAGRAM statement can be used to request a diagram plot, and the parameter estimates can be shown in either standardized or unstandardized units. In factor analysis, standardized factor loadings are correlations. For example, $\rho \left( SGST,{F}_1\right)=\frac{\lambda_{11} Var\left({F}_1\right)}{\sqrt{Var\left({F}_1\right) Var(SGST)}}=\lambda \sqrt{\frac{Var\left({F}_1\right)}{Var(SGST)}}$. But in MIMIC model, the standardized factor loadings are partial correlations between the latent structure and the indicators controlling for the exogenous variables in the model. The variances are derived from *Cov*(***Y***) **= Λ**(**ΓΘ**_***X***_**Γ**^′^ ***+*** **Ψ**)**Λ**^′^ ***+*** **Θ**_***ϵ***_, *Cov*(***η***) = **ΓΘ**_***X***_**Γ**^′^ ***+*** **Ψ**.

Similarly, standardized variances and covariances of exogenous variables (i.e. **X**) are also correlations derived from **Θ**_***X***_. However, standardized variances and covariances among errors *ϵ* and disturbances *ζ* are calculated as ${\theta}_{ij}^{\ast }=\frac{\theta_{ij}}{\sqrt{\sigma_{ii}^2{\sigma}_{jj}^2}}$ for two terms (*i*, *j*) where (*θ*_*ij*_) is the covariance and ${\sigma}_{ii}^2$ is the variance of the outcome corresponding to term *i*. The standardized variance is ${\theta}_{ii}^{\ast }=\frac{\theta_{ii}}{\sigma_{ii}^2}$. Hence, ${\theta}_{ij}^{\ast }$ is not a correlation, and ${\theta}_{ii}^{\ast }$ does not necessarily equal 1. By default, ${\sigma}_{ii}^2$ called the total variance is reported for **Y** and ***η***.

**Fig. 4:**
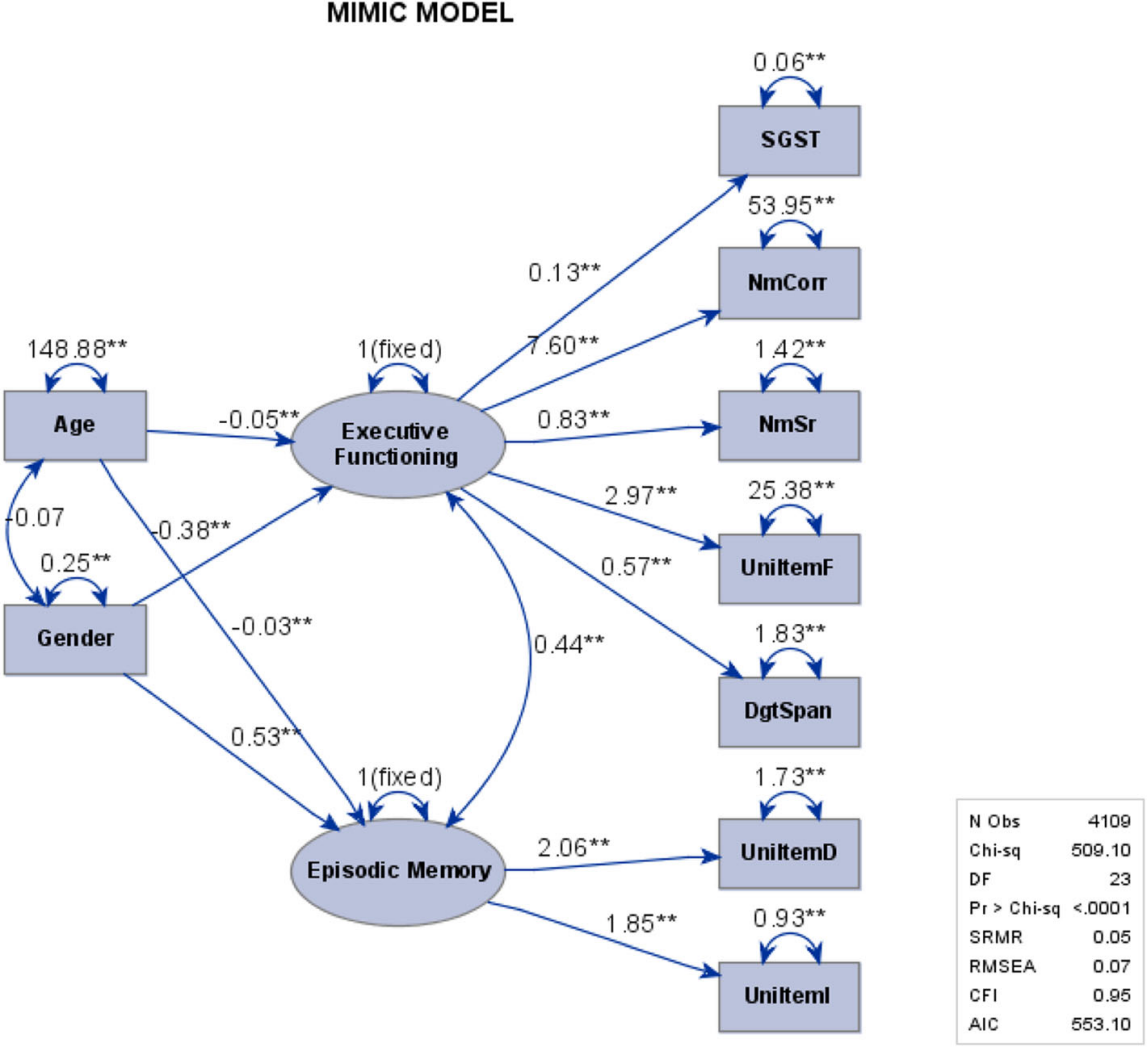
SAS Diagram generated from the pathdiagram statement in SAS syntax

In our example SAS syntax, the diagram with unstandardized estimate was specified. One can replace it with “diagram = [initial unstandard standard]” to generate three diagrams available: the diagram with initial framework, the diagram with unstandardized estimate, and the diagram with standardized estimate. Noted that the additional option “exogcov” has to be specified right after the type of the diagram to have the covariance of exogenous variables shown on the diagram. Also, when the exogcov is specified, only one type of diagram can be generated. For simplicity, we only showed the diagram with unstandardized estimate for the demonstration purpose across three statistical software packages. Also, we only requested four commonly-seen indices in the plot for the purpose of simplicity. By default, SAS outputs seven absolute indices, nine parsimony indices, and six incremental indices (Fig. 4).

##### Fit statistics

Our model is estimated by maximum likelihood (ML). A likelihood ratio statistic comparing the fitted model (with 22 parameters) to the unconstrained saturated model with 45 parameters produces has a *χ*^2^ statistic of 509.1 with df = 23. The *χ*^2^ test is significant suggesting poor fit. However, this is often the case with large sample size. ML estimation is based of minimizing the objective function *OBF* = *trace*(***S*Σ**^***−*1**^) ***−*** (*p* + *q*) =  *log* (|**Σ**|) ***−***  *log* (|***S***|)***.*** The *χ*^2^ statistic is (*N* − 1) times the minimized objective function. Hence when the sample size *N* is large, the *χ*^2^ test is likely to be significant. Standardized root mean square residual (SRMR), Bentler Comparative Fit Index (CFI) are used to assess model fit. In this example, SRMR = .047 and CFI = .952, which are near the recommended cutoffs of < .08 and > .95, indicating that the model is a good-fitting model. Comparative indices, such as AIC and BIC, are used to compare competing models, that is, with different covariance structures. Since there is no competitive model in our example, AIC is not used in this case.

#### M*plus*

**Fig. 5:**
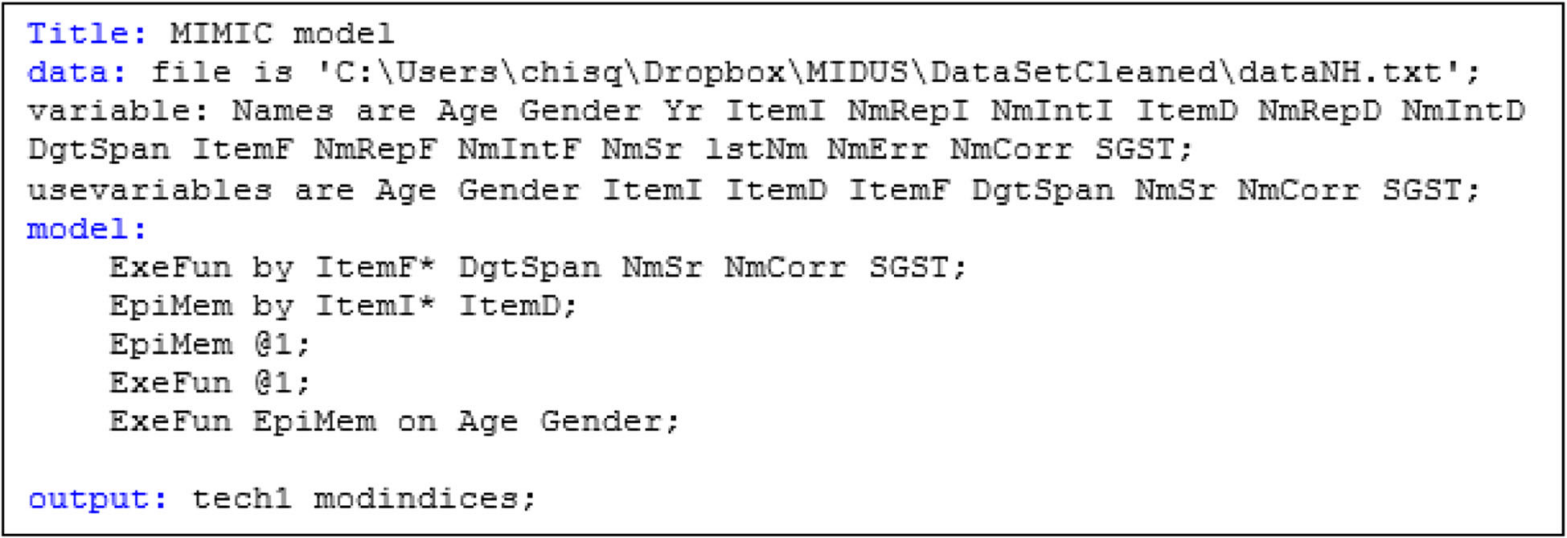
M*plus* input code for the MIMIC model

M*plus* is designed specifically for latent variable modeling, which is commonly used in social science and psychology to examine latent variable framework. M*plus* will save a .dgm file every time it runs. Researchers can click the Diagram tab on the tool bar, and then choose ‘Open Diagrammer’ to open the M*plus* Diagram module. The latent constructs, estimates, and indicators in the diagram can be repositioned by clicking and dragging (Fig. 5).

**Fig. 6:**
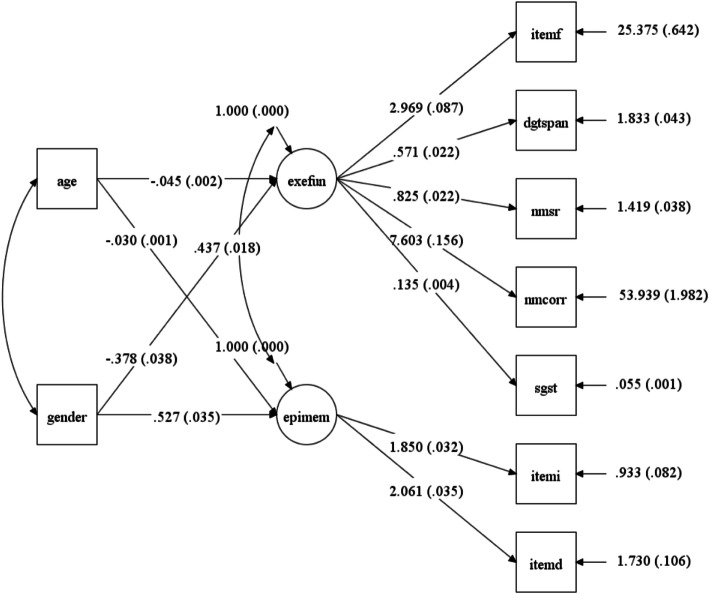
M*plus* Diagram for the MIMIC model

M*plus* input consists of four sections: title, data, model, and output. The detailed options and choices for M*plus* can be seen in its user guide [18]. Both raw data and covariance matrix data can be the input data. The model is specified in the model section. The ‘by’ statement is used to specify measurement model (see equation (1)). The term specified before the by statement is the name of the latent construct (*η*), and the variables specified after the by statement are the indicators (Y) that were used to measure the latent construct. The sign “@” is used to fix the parameter estimation. The statement “EpiMem @ 1” means we would like to fix the residual of the latent construct EpiMem at 1. Since EpiMem and ExeFun are the latent variables named by the researchers, the statements of fixing the residual variances at 1 have to be specified after the measurement model; otherwise, M*plus* would not be able to know ExeFun and EpiMem are the latent constructs. The last command in the model section “ExeFun EpiMem on Age Gender” is to specify the structure model (see equation (2)), indicating the regression of latent constructs ExeFun and EpiMem on Age and Gender, or predicting ExeFun and EpiMem from gender and age (Fig. 6).

M*plus* output provides five sections (see Supporting Information): 1) model specification, 2) univariate sample statistics, 3) model fit information, 4) model results, and 5) model modification indices. The first and second sections are provided for the researchers to verify that the model they specified in the input command and the data they read in are correct. M*plus* is mainly designed for running latent variable models and has limited data manipulation functionality. Researchers are advised to clean the data before using the software. More details about M*plus*, syntax and examples can be found on the website: statmodel.com.

#### R

**Fig. 7:**
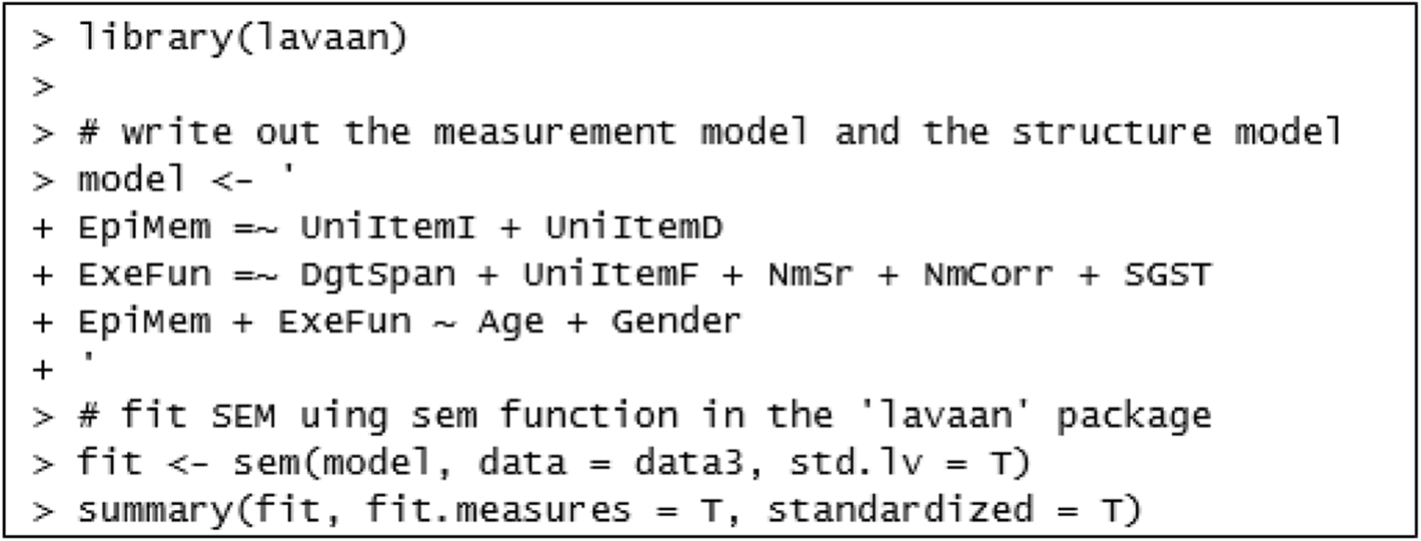
R code for the MIMIC model

R packages *lavaan* and *semPlot* are used in this paper. The former is used to run structural equation modeling, and the latter one is for generating the diagram. The format of the R output is very similar to M*plus* output (Fig. 7).

In the model results (output is shown in the Supporting Information), two extra columns of the parameter estimates are printed at the end of the table. The Std.lv column reported the estimates when the latent variables “EpiMem” (Episodic Memory) and “ExeFun” (Executive Function) were standardized. The last column Std.all reported the parameter estimates when both the latent variables and the observed variables were standardized (also called the ‘completely standardized solution’).

**Fig. 8:**
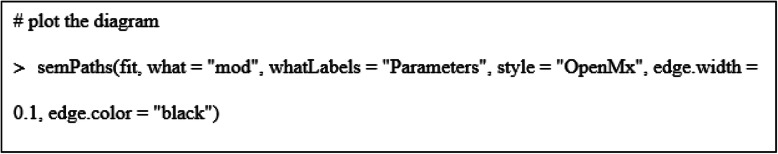
R code for the MIMIC model diagram

The following code can be used to generate the diagram for the proposed model (Fig. 8).

**Fig. 9:**
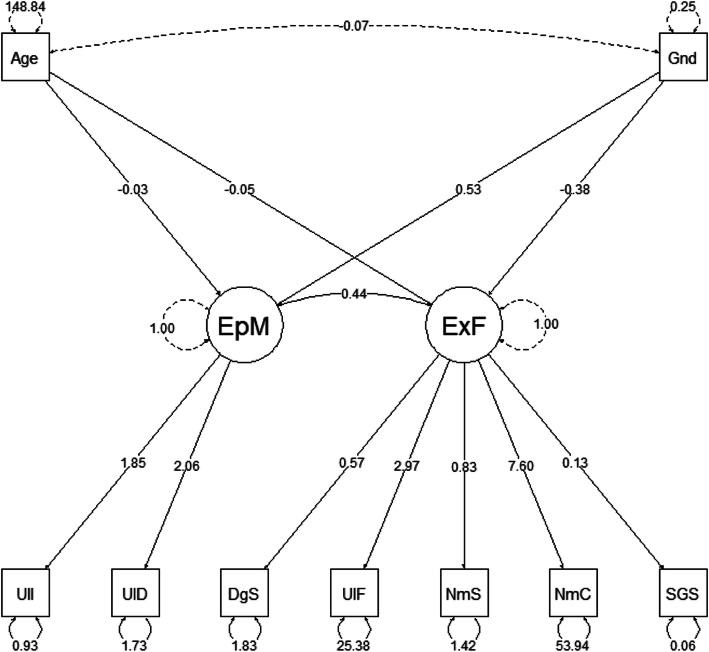
the MIMIC model Diagram from R *semPlot* package

The function *semPaths* is used to plot the SEM diagram. The solid arrow lines in the diagram are the 19 model parameters. The remaining three parameters shown as dashed arrow lines are 1) the residual variances of the latent constructs, which are fixed at 1 for scale indeterminancy, and 2) the covariance between age and gender that is given by the covariance matrix from the data set. Because the variance and covariance of the predictors are not model parameters and can be obtained from the covariance matrix, they were shown as dashed arrow lines in the diagram in R, for researchers’ information. Also, since the residual variances of the latent constructs are fixed at 1, they are shown as dashed arrows as well (Fig. 9).

#### Parameter estimates comparison

The results in Table 2 show the statistically significant model parameter estimates. Estimates from three statistical software packages are almost exactly the same. The differences are very trivial. The t test statistics of the parameter estimates across three packages are identical. For simplicity, only t-value of SAS results are shown in Table 2. In this example, we provided evidence that age and gender can contextualize the cognitive functioning performance. Specifically, age and gender are significantly associated with each latent construct of cognitive functioning performance. Men have better scores on executive function compared to women, while women have better scores than men on episodic memory. The correlation between executive function and episodic memory of 0.44 is significant, but the covariance between age and gender is not (so is not included in the table).

**Table 2:** Effects in Linear Equations and Estimates of the Variances – MIMIC model

Parameter	Indicators	Latent Variable	Parameter	SAS	M*plus*	R	t-value (SAS)
Factor Loading	SGST (Y1)	Executive Functioning	*λ*_11_	0.13 (0.004)	0.14 (0.004)	0.14 (0.004)	33.53
	NmCorr (Y2)	Executive Functioning	*λ*_21_	7.60 (0.159)	7.60 (0.159)	7.60 (0.159)	47.8
	NmSr (Y3)	Executive Functioning	*λ*_31_	0.83 (0.021)	0.83 (0.022)	0.83 (0.021)	38.49
	UniItemF (Y4)	Executive Functioning	*λ*_41_	2.97 (0.087)	2.97 (0.087)	2.97 (0.087)	34.24
	DgtSpan (Y5)	Executive Functioning	*λ*_51_	0.57 (0.022)	0.57 (0.022)	0.57 (0.022)	26.01
	UniItemD (Y6)	Episodic Memory	*λ*_62_	2.06 (0.036)	2.06 (0.035)	2.06 (0.036)	57.84
	UniItemI (Y7)	Episodic Memory	*λ*_72_	1.85 (0.031)	1.85 (0.032)	1.85 (0.031)	59.15
Regression Coefficient	Age	Executive Functioning	*γ*_11_	−0.05 (0.002)	−0.05 (0.002)	−0.05 (0.002)	−26.8
	Gender	Executive Functioning	*γ*_12_	−0.38 (0.038)	−0.38 (0.038)	− 0.38 (0.038)	−10
	Age	Episodic Memory	*γ*_21_	−0.03 (0.001)	−0.03 (0.001)	− 0.03 (0.001)	−20.73
	Gender	Episodic Memory	*γ*_22_	0.53 (0.035)	0.53 (0.035)	0.53 (0.034)	15.27
Residual Variance	SGST (Y1)		${\sigma}_{\epsilon_1}^2$	0.06 (0.001)	0.06 (0.001)	0.06 (0.001)	39.96
	NmCorr (Y2)		${\sigma}_{\epsilon_2}^2$	53.95 (1.944)	53.95 (1.982)	53.95 (1.943)	27.75
	NmSr (Y3)		${\sigma}_{\epsilon_3}^2$	1.42 (0.038)	1.42 (0.038)	1.42 (0.038)	37.4
	UniItemF (Y4)		${\sigma}_{\epsilon_4}^2$	25.38 (0.640)	25.38 (0.642)	25.38 (0.640)	39.65
	DgtSpan (Y5)		${\sigma}_{\epsilon_5}^2$	1.83 (0.043)	1.83 (0.043)	1.83 (0.043)	42.45
	UniItemD (Y6)		${\sigma}_{\epsilon_6}^2$	1.73 (0.106)	1.73 (0.106)	1.73 (0.106)	16.39
	UniItemI (Y7)		${\sigma}_{\epsilon_7}^2$	0.93 (0.082)	0.93 (0.082)	0.93 (0.082)	11.39
Covariance		Executive Functioning vs. Episodic Memory	*cov*(*ζ*_1_, *ζ*_2_)	0.44 (0.017)	0.44 (0.018)	0.44 (0.017)	25.3

## Discussion

This paper aimed to provide a tutorial guideline to conducting the MIMIC model using SAS CALIS procedure, M*plus*, and R *lavaan* package. We first provide the introduction of the model under LVM framework, then demonstrated the input commands for conducting the MIMIC model, generated diagrams with the three commonly used statistical software packages and illustrated the results and diagrams of the model.

The method section of this paper elaborated how the MIMIC model is specified and identified. We can see that a MIMIC model is a combination of path analysis and factor analysis, and that from a different perspective it can be seen as path analysis with latent-constructed outcome variables where the measurement errors are considered. The model has the following features. First, it is a one-step solution incorporating multiple indicators (the measurement model of SEM) and multiple causes (the structural model of SEM), while at the same time managing the inflated type I error rate that may arise from multiple testing. Second, the model can be utilized as a psychometric evaluation technique for differential item functioning (DIF) [8–10], measurement invariance [19], multiple-group analysis [10, 11], and multidimensional measures [20]. Third, the factor scores extracted from the MIMIC model are the conditioned factor scores controlled for the demographic variables.

The MIMIC model is also referred to as factor analysis with covariates. Similarly, as a special case of SEM and also under the LVM framework, when the latent variables in the model are categorical, it is called a latent class analysis (LCA) with covariates, or latent class model with one-step procedure. Like the covariates in the MIMIC model, covariates in LCA have the same role of contextualizing the latent variables. Chang [21] extended the LCA model in multilevel contexts (e.g., students in after school programs) and used predictors at the student level and program level to contextualize the latent constructs. Similarly, future studies could extend MIMIC model to multilevel contexts or longitudinal scenarios. The transition of a subject on the latent factor (e.g., quality of life) at different time points can be modelled before and after an intervention. Meanwhile, the characteristics of groups of subjects with growth trajectories can be identified by incorporating their covariates in the model. In biostatistics, the path analysis part of MIMIC models can be extended to allow the indicators to be latent variables [22].

## Conclusions

In this paper, we provided input code of three statistical software packages: SAS, M*plus*, and R. Interpretation of the output and diagrams were also provided. We examined the effect of age and gender on cognitive function using 4109 participants in MIDUS II dataset. The results found that there is a significant gender disparity in cognitive functioning controlling for their age. Males tend to have better scores on executive functioning compared to females, while females have better scores on episodic memory compared to males. This result replicated Lechman et al’s study by fitting the data in the MIMIC model. The MIMIC model introduced in this paper incorporated the covariates of interest in the factor analysis, making the statistical modeling more approachable, the fitting procedure easier, and the results more rigorous. Since results were identical across three statistical software packages, application researchers can focus on constructing the MIMIC model of interest and the theoretical framework without concerning which software package should be used.

For simplicity, we used standard maximum likelihood estimation for the MIMIC model in this study since the sample size is large and the variables are continuous. All three statistical programs introduced in this study, the CALIS procedure in SAS, M*plus*, and R *lavaan* package, have numerous estimation options available, such as maximum likelihood with robust standard error. Application researchers can choose an appropriate estimation method according to the type of the variables and dataset. More details about the performance of different estimation methods in latent variable models can be found in Li’s study [23].

The MIMIC model has been commonly utilized in applied research [24–26] but is mainly confined to the disciplines of mental health, social science, and education previously. In this paper, we provided syntax in three commonly used statistical software packages, explanation of the MIMIC model, and an empirical application to a real data set. We hope this paper can serve as a tutorial of MIMIC model and help facilitate the process of rigorous research for applied researchers in a diversity of fields.

## Data Availability

The datasets analyzed during the current study are available in the Inter-university Consortium for Political and Social Research repository, https://www.icpsr.umich.edu/icpsrweb/ICPSR/studies/25281 Ryff CD, Lachman ME. Midlife in the United States (MIDUS 2): Cognitive Project, 2004–2006. 2017. doi:10.3886/ICPSR25281.v6

## Abbreviations

SEM: Structural Equation Modeling
LVM: Latent Variable Modeling
MIMIC model: multiple-indicator, multiple-cause model
MIDUS II: Midlife in the United States II
SGST: Stop and Go Switch Task (SGST)
NmCorr: 30 Seconds and Counting Task
NmSr: Number Series
UniItemF: Category Verbal Fluency
DgtSpan: Backward Digit Span
UniItemD: Delayed Word List Recall
UniItemI: Immediate Word List Recall
CFA: confirmatory factor analysis

## References

[1] Ryff CD, Lachman ME (2017). Midlife in the United States (MIDUS 2): cognitive project, 2004–2006.

[2] Todd D. Little. The Oxford handbook of quantitative methods. Oxford Libr Psychol. 2013;2: Statist:551-. doi:10.1017/CBO9781107415324.004.

[3] Lachman ME, Agrigoroaei S, Tun PA, Weaver SL (2014). Monitoring cognitive functioning: psychometric properties of the brief test of adult cognition by telephone. Assessment..

[4] Bollen KA, Long JS. Testing Structural Equation Models. Newbury Park: Sage Publications; 1993.

[5] Jöreskog KG, Goldberger AS. Estimation of a model with multiple indicators and multiple causes of a single latent variable. J Am Stat Assoc. 1975;70(351):631–639.

[6] O’Rourke N, Hatcher L. A Step-by-Step-Approach to Using SAS for Factor Analysis and Structural Equation Modeling. 2nd Ed. Cary, NC: SAS Institute; 2013.

[7] Wang J, Wang X. Structural equation modeling: applications using M*plus*. Hoboken: Wiley; 2012.

[8] Finch H. The MIMIC model as a method for detecting DIF. 2005;29(4):278–295. doi:10.1177/0146621605275728.

[9] Woods CM, Grimm KJ (2011). Testing for nonuniform differential item functioning with multiple indicator multiple cause models. Appl Psychol Meas.

[10] Woods CM (2009). Evaluation of MIMIC-model methods for DIF testing with comparison to two-group analysis. Multivariate Behav Res.

[11] Raykov T, Marcoulides GA, Lee C-L, Chang C (2013). Studying differential item functioning via latent variable modeling: a note on a multiple-testing procedure. Educ Psychol Meas.

[12] Jöreskog KG, van Thillo M. LISREL: A general computer program for estimating a linear structural equation system involving multiple indicators of unmeasured variables. Princeton: Educational Testing Servicem; 1972.

[13] Kaplan D. Structural equation modeling. Sage Publications, Inc; 2000.

[14] Kenny DA. Measuring Model Fit. http://davidakenny.net/cm/fit.htm. Published 2020.

[15] Hooper D, Coughlan J, Mullen MR (2008). Structural equation modelling: Guidelines for determining model fit. Electron J Bus Res Methods.

[16] Bentler PM, Bonett DG (1980). Significance tests and goodness of fit in the analysis of covariance structures. Psychol Bull.

[17] Bentler PM (1990). Comparative fit indexes in structural models. Psychol Bull.

[18] Muthén LK, Muthén BO. Mplus User’s Guide. 8th Ed. Los Angeles: Muthén & Muthén; 2018.

[19] Masyn KE (2017). Measurement invariance and differential item functioning in latent class analysis with stepwise multiple indicator multiple cause modeling. Struct Equ Model.

[20] Lee S, Bulut O, Suh Y (2017). Multidimensional extension of multiple indicators multiple causes models to detect DIF. Educ Psychol Meas.

[21] Chang C (2016). Nonparametric multilevel latent class analysis with covariates: an approach to classification in multilevel contexts [dissertation].

[22] Tekwe CD, Zoh RS, Bazer FW, Wu G, Carroll RJ (2018). Functional multiple indicators, multiple causes measurement error models. Biometrics..

[23] Li CH (2016). Confirmatory factor analysis with ordinal data: comparing robust maximum likelihood and diagonally weighted least squares. Behav Res Methods.

[24] Guan M. Measuring the effects of socioeconomic factors on mental health among migrants in urban China: a multiple indicators multiple causes model. Int J Ment Health Syst 2017;11(1):1–12. doi:10.1186/s13033-016-0118-y. 10.1186/s13033-016-0118-y PMC5217273 28070220

[25] Proitsi P, Hamilton G, Tsolaki M (2011). A multiple indicators multiple causes (MIMIC) model of behavioural and psychological symptoms in dementia (BPSD). Neurobiol Aging.

[26] Brailean A, Guerra M, Chua KC, Prince M, Prina MA (2015). A multiple indicators multiple causes model of late-life depression in Latin American countries. J Affect Disord.

